# Longitudinal Integrated Foundation Training: uplifting perspectives

**DOI:** 10.1111/medu.13709

**Published:** 2018-10-21

**Authors:** Kathryn E Burnett, Catherine Tregoning, David A Hirsh, Paul Baker

## What problems were addressed?

In the UK, upon completion of undergraduate medical training, doctors enter postgraduate training in a generic 2‐year programme called the Foundation Programme (FP). In the FP, trainees typically rotate through six 4‐month clinical placements. Five placements normally occur in the secondary care environment and one in a primary care setting, commonly in general practice (GP). Trainees rotate (change venues) frequently, have short times to accommodate to the dynamic work environments, and experience difficulties in building rich relationships with patients, supervisors and the caring team. Trainees also find it challenging to access support. For some trainees, the timing of their GP placement comes after they have had to make choices around specialty training. Others find the experience insufficient to enable them to make an informed career choice.

## What was tried?

In August 2016 the North West of England Foundation School (NWoEFS) began its pilot of Longitudinal Integrated Foundation Training (LIFT), based upon the success of Harvard Medical School's Cambridge Integrated Clerkship. The LIFT initiative aims to animate the best of an apprenticeship model. This programme provides more exposure to primary care over the 2‐year period. It was designed to address the contextual difficulties found in traditional rotations, to enhance trainees’ transferable clinical and communication skills and to foster trainees’ understanding of the National Health Service. Trainees spend 1.5 days each week within the same GP setting for the 2 years. During the remaining 3.5 days, they rotate every 4 months around hospital specialties as per traditional FP training. Under this longitudinal model, the GP placement provides educational continuity.

To date, 47 trainees and their supervisors have engaged in LIFT through a full 2‐year FP training cycle. Researchers at NWoEFS are evaluating LIFT through interviews, focus group discussions and online surveys. Trainees and supervisors alike have required information and support to adapt to the scheme. Coordinating the rota has facilitated concurrent working across primary and secondary care. The leaders have identified and worked to address logistical and financial needs, especially around backfill of ‘lost’ hospital time and supply of GP supervisors.

## What lessons were learned?

We have analysed trainees’ and supervisors’ perspectives. As the pilot nears its conclusion, many supervisors have expressed the hope that LIFT continues. Supervisors feel that the programme overcame challenges experienced during the initial phase (practicalities around rotas, travel between primary and secondary care settings and handovers) and that it has numerous advantages. Supervisors consider LIFT trainees to often have greater clinical knowledge, independent decision‐making skills, greater patient‐centredness and improved ability to take a holistic approach to patients and their care in comparison with their non‐LIFT counterparts. Supervisors have built strong and effective relationships with trainees, offer better pastoral as well as educational support, and over time have built sufficient connections with trainees to be able to offer increased challenge to them. Trainees who have participated in LIFT have demonstrated that although initially they felt less experienced in secondary care than peers working a 5‐day hospital week, as anticipated by supervisors, they have become aligned with non‐LIFT trainees or even surpassed them. The pilot has generated practical lessons for future education and care delivery.^1^
